# Supervised Machine Learning-Based Prediction of In-Hospital Mortality Following Hip Fracture in Older Adults

**DOI:** 10.3390/diagnostics16040612

**Published:** 2026-02-19

**Authors:** Eduardo Guzmán-Muñoz, Manuel Vásquez-Muñoz, Yeny Concha-Cisternas, Rodrigo Olivares-Ordenes, Vicente Clemente-Suárez, Antonio Castillo-Paredes, Rodrigo Yáñez-Sepúlveda

**Affiliations:** 1 Escuela de Kinesiología, Facultad de Salud, Universidad Santo Tomás, Talca 3460000, Chile; eguzmanm@santotomas.cl; 2 Escuela de Pedagogía en Educación Física, Facultad de Educación, Universidad Autónoma de Chile, Talca 3460000, Chile; 3 Center for Health Data Observation and Analysis (CADS), School of Medicine and Health Sciences, Universidad Mayor, Santiago 8580745, Chile; 4 Escuela de Medicina, Facultad de Medicina y Ciencias de la Salud, Universidad Mayor, Santiago 8580745, Chile; 5 Vicerrectoría de Investigación e Innovación, Universidad Arturo Prat, Iquique 1100000, Chile; 6 Escuela de Ingeniería Informática, Universidad de Valparaíso, Valparaíso 2340000, Chile; rodrigo.olivares@uv.cl; 7 Faculty of Medicine, Universidad Europea de Madrid, 28670 Madrid, Spain; vctxente@yahoo.es; 8 Grupo de Investigación en Cultura, Educación y Sociedad, Universidad de la Costa, Barranquilla 080002, Colombia; 9 Grupo AFySE (Actividad Física y Salud Escolar), Escuela de Pedagogía en Educación Física, Facultad de Educación, Universidad de Las Américas, Santiago 8370040, Chile; acastillop85@gmail.com; 10 Facultad de Educación y Ciencias Sociales, Universidad Andrés Bello, Viña del Mar 2200055, Chile; rodrigo.yanez.s@unab.cl; 11 School of Medicine, Universidad Espíritu Santo, Samborondón 092301, Ecuador

**Keywords:** hip fracture, older adults, in-hospital mortality, machine learning, Gradient Boosting, SHAP analysis, predictive modeling, explainable AI

## Abstract

**Background/Objectives:** Hip fractures in older adults are associated with substantial morbidity, functional decline, and high in-hospital mortality. Early identification of patients at increased risk of death may improve clinical decision-making and resource allocation. This study aimed to develop and internally validate supervised machine learning models to predict in-hospital mortality among older adults hospitalized for hip fracture using nationwide administrative data from Chile. **Methods:** A retrospective cohort study was conducted using anonymized hospital discharge records from the Chilean National Health Fund (FONASA), covering admissions between 1 January 2019 and 31 December 2024, across 72 public hospitals. Demographic, clinical, and care-related variables were included as predictors. Multiple supervised machine learning algorithms were trained and evaluated using stratified train–test partitioning. Model performance was assessed using AUC-ROC, precision, recall, and F1-score. Model interpretability was explored using SHapley Additive exPlanations (SHAP). **Results:** A total of 40,253 hospitalization episodes were analyzed. The Gradient Boosting model achieved the best overall performance, with an AUC-ROC of 0.885 and a favorable balance between precision and recall. SHAP analysis identified age, comorbidity burden, and surgical treatment as the most influential predictors, revealing nonlinear and clinically meaningful contributions to mortality risk. **Conclusions:** Supervised machine learning models based on routinely collected administrative data demonstrated strong predictive performance for in-hospital mortality after hip fracture. Interpretable models may support early risk stratification and clinical decision-making at a national healthcare level.

## 1. Introduction

Hip fracture among older adults remains a pressing global health issue, affecting approximately 1.5 million people each year, with projections suggesting this figure could rise to 2.6 million by 2025 and 4.5 million by 2050 [1]. In 2019, the global incidence in individuals aged 55 years and older was 681.35 per 100,000 population, with a prevalence of 1191.39 and a burden of 130.78 years lived with disability (YLDs) per 100,000 [2]. Over the past three decades, incidence rates have declined among those under 60 but increased sharply in older adults, particularly in women. Although rates remain consistently higher in females, the male-to-female incidence ratio rose modestly from 0.577 to 0.612 between 1990 and 2019 [2]. Falls continue to be the predominant cause across all age groups. These trends highlight the escalating burden of hip fractures in aging populations and the urgent need for improved prevention and prognostic strategies.

These injuries have profound clinical and functional consequences. Hip fractures often lead to loss of mobility, reduced independence, and a substantial decline in quality of life [3,4]. Mortality rates remain high despite advances in surgical and perioperative care—approximately 2–15% of patients die during hospitalization [5,6,7,8], and 10–30% within the first year after the event [9,10]. Although advances in surgical management and perioperative care have improved clinical practice, in-hospital mortality after hip fracture remains substantial. Identifying individuals at increased risk of in-hospital death is therefore essential for optimizing clinical pathways and ensuring the most efficient allocation of healthcare resources. As populations age and the incidence of hip fractures continues to rise, the development of accurate, transparent, and clinically meaningful prognostic models becomes increasingly important to support evidence-based decision-making [11].

This challenge is particularly relevant in Chile, where the burden of hip fractures among older adults has grown sharply over recent years. National hospital discharge data indicate that admissions increased by nearly 50% between 2006 and 2017, reaching about 9583 cases—equivalent to 149 per 100,000 adults aged 45 years and older [12]. The direct cost of managing these injuries exceeds 34 million USD annually, placing a considerable strain on the national healthcare system [12]. Hip fractures, therefore, represent a major clinical and public health concern in Chile. Predictive modeling based on routinely collected hospital information may help clarify mortality patterns and inform more effective prevention and management strategies.

In recent years, the use of machine learning in clinical research has expanded rapidly, offering new possibilities for predicting outcomes in complex and heterogeneous patient populations. In contrast to traditional regression models that rely on predefined linear assumptions, machine learning algorithms are capable of capturing nonlinear interactions and identifying multidimensional patterns within data, thereby improving the accuracy of prognostic estimations [13]. A wide range of supervised machine learning techniques—including tree-based ensemble methods, support vector machines, neural networks, and others—have been applied to prognosis in surgical and geriatric medicine [11,14]. Importantly, recent advances in geriatric risk prediction have increasingly emphasized not only predictive performance but also interpretability and clinical usability through explainable artificial intelligence approaches (e.g., SHAP) and user-oriented risk tools, as illustrated by a recent cohort study that developed a visualized machine learning system for sarcopenia risk prediction in older adults [15].

In the field of hip fracture research, several studies have begun incorporating machine learning–based models to estimate mortality risk [16,17,18]. Some of these approaches have achieved predictive performance comparable to, or even exceeding, that of conventional regression models. For example, a machine learning algorithm based on gradient-boosted decision trees achieved an AUC of about 0.79 for predicting 30-day mortality using preoperative clinical variables in a large dataset [13]. Similarly, random forest and naïve Bayes classifiers have shown comparable accuracy to logistic regression in predicting in-hospital and short-term mortality following hip fracture [11]. However, most prior studies focus on a single modeling technique; there is a relative paucity of research directly comparing multiple machine learning algorithms in this setting [19].

To address this gap, the present study aimed to develop and internally validate several supervised machine learning models for predicting in-hospital mortality among older adults with hip fracture, using routinely collected national hospital data from Chile. We hypothesized that supervised machine learning models—particularly tree-based ensemble methods—would provide accurate and clinically interpretable predictions of in-hospital mortality after hip fracture using routinely collected hospital data, outperforming traditional linear approaches.

## 2. Materials and Methods

### 2.1. Study Design and Data Source

This retrospective cohort study developed and internally validated supervised machine learning models to predict in-hospital mortality among older adults hospitalized for hip fracture in Chile. The analysis used anonymized hospital discharge data from the Chilean National Health Fund (Fondo Nacional de Salud, FONASA), which consolidates standardized administrative records from the 72 public hospitals forming the national healthcare network. These institutions deliver moderate- to high-complexity care, including orthopedic trauma and surgical management. The database contains detailed information on patient demographics, diagnostic and procedural codes, and discharge outcomes. Data were extracted from the open-access FONASA repository (https://datosabiertos.fonasa.cl, accessed on 9 February 2026) and included all hospitalizations between 1 January 2019, and 31 December 2024. Each individual was identified using an encrypted unique patient identifier, which allowed linkage of multiple hospitalizations belonging to the same patient. To avoid within-patient clustering and ensure independence of observations, only the first hospitalization for hip fracture per individual during the study period was retained for analysis.

FONASA covers roughly 85% of Chileans aged 60 years and older, representing the public healthcare sector where most hip fractures occur. The dataset undergoes regular audits by the Ministry of Health to ensure internal consistency and coding accuracy. All stages of model development and validation adhered to the TRIPOD-AI reporting guideline, ensuring transparency and reproducibility in the construction of AI-based clinical prediction models.

### 2.2. Participants

All hospitalizations of adults aged 60 years and older with a principal diagnosis of hip fracture were included. Cases were identified according to the International Classification of Diseases, Tenth Revision (ICD-10) codes: S72.0 (femoral neck fracture), S72.1 (pertrochanteric fracture), and S72.2 (subtrochanteric fracture). Exclusion criteria comprised: (i) missing information on age, sex, or discharge status; (ii) diagnostic codes not falling within the predefined range; (iii) transfer cases or duplicate admissions; and (iv) records with all predictor variables absent. Exclusions were intentionally restricted to data quality–related issues in order to preserve the representativeness of the national cohort. Clinical variables not captured in administrative records—such as physiological parameters, laboratory values, time to surgery, or frailty scales—were not excluded but were unavailable in the dataset.

To ensure data integrity, validation procedures involved cross-checking unique admission and discharge identifiers to confirm internal consistency and remove duplicates. The final analytic cohort included 40,253 hospitalization episodes of older adults treated across the 72 public hospitals in Chile.

### 2.3. Outcome

The primary outcome was in-hospital mortality, defined as death occurring during the same hospitalization period. Mortality status was extracted from the standardized discharge condition field within the FONASA database, which documents each patient’s vital status at discharge.

During data preprocessing, the positive class (death) was identified automatically through string matching for terms such as “falle”, “muer”, “defun”, and “dead” appearing in the discharge condition variable. When no direct match was detected, the final label was assigned lexicographically to preserve consistency across the dataset. For clinical coherence and interpretability, death was pre-specified as the positive outcome class in both sensitivity and main analyses.

Only deaths verified at the time of discharge were included; post-discharge mortality data were not available. Each record corresponded to a distinct hospitalization episode, confirmed through cross-referencing of admission and discharge identifiers to avoid duplication.

### 2.4. Predictors

Predictor variables were selected a priori based on three predefined criteria: (i) consistent availability across the national administrative hospital discharge database, (ii) early availability during hospitalization to allow potential clinical applicability, and (iii) prior evidence supporting their association with in-hospital mortality after hip fracture. These criteria were applied to ensure both methodological robustness and clinical relevance while preserving the generalizability of the model to real-world inpatient settings. Based on these criteria, seven routinely collected variables were selected for model development and organized into three conceptual domains:

1. Demographic variables: Sex (male/female), ethnicity (categorical), and age (continuous, in years) [5,6,7,8].
2. Disease burden and treatment: Number of comorbidities (count of secondary diagnoses recorded at discharge) and surgical treatment (yes/no) [5,6,7,8].
3. Care process indicators: Transferring service or first hospital ward (categorical; e.g., orthopedics, internal medicine, intensive care) and length of hospital stay (continuous, in days) [5,6,7,8].

Categorical variables were encoded using one-hot encoding, while continuous variables were standardized before model training. This preprocessing step facilitated comparability among predictors and minimized potential bias related to variable scaling during model optimization. All selected predictors were available for all patients prior to discharge, allowing model application in early inpatient settings.

### 2.5. Data Management and Preprocessing

To ensure robustness and reproducibility across heterogeneous CSV exports, a standardized data management and preprocessing workflow was established. The datasets were imported through an automated parser capable of recognizing multiple delimiters (;, ,, \t, |) and character encodings (UTF-8 or Latin-1). Lines containing formatting inconsistencies were automatically omitted to preserve dataset integrity. Column headers were normalized to lower-case ASCII and converted to snake_case notation, while synonym mapping was applied to standardize variable names. For instance, variables such as “cirugia_(0 = no/1 = si)” were unified as “cirugia,” and all variations referring to “days of hospitalization” were consolidated under a single label.

Before model training, variable types were harmonized to ensure compatibility across models. The variable representing surgical treatment was recoded to binary format (0/1, yes/no, or true/false), and both age and length of stay were converted to numerical type. Records lacking outcome data or missing all predictor values were removed. Missing data were handled by imputing the median for continuous variables and the mode for categorical ones. Numerical features were scaled, and categorical variables were encoded using a OneHotEncoder configured to ignore unseen categories. Both transformations were implemented within a ColumnTransformer to ensure consistent preprocessing across datasets.

To prevent data leakage, all preprocessing steps were embedded within the machine learning pipeline using scikit-learn and fitted exclusively on the training data. This design safeguarded methodological transparency, reproducibility, and the validity of model evaluation.

### 2.6. Data Partition and Validation Strategy

The dataset was randomly divided into training and testing subsets using an 80/20 stratified split to maintain the original distribution of the outcome classes (alive vs. deceased). Randomization was performed with a fixed seed (random_state = 42) to allow full reproducibility of the results. Each hospitalization episode was assigned to only one subset, ensuring that no records overlapped between the training and testing datasets and thereby preserving a strict separation throughout model development and evaluation.

### 2.7. Model Development

Eight supervised classification algorithms were developed and trained under a standardized scikit-learn framework to enable consistent comparison across models. The classifiers evaluated were:

1. Naïve Bayes (GaussianNB),
2. Multilayer Perceptron (MLP) neural network with two hidden layers (64 and 32 neurons, ReLU activation, α = 1 × 10^−3^, max_iter = 500, random_state = 42),
3. Random Forest (400 estimators, class_weight = “balanced_subsample”, n_jobs = –1),
4. Gradient Boosting (default parameters, random_state = 42),
5. k-Nearest Neighbors (kNN) (*k* = 7, distance-weighted voting),
6. Logistic Regression (solver = LBFGS, max_iter = 1000, class_weight = “balanced”),
7. Support Vector Machine (SVM) with radial basis function (RBF) kernel (*C* = 2.0, gamma = “scale”, probability = True, class_weight = “balanced”, random_state = 42), and
8. Decision Tree (class_weight = “balanced”).

All algorithms were implemented as scikit-learn Pipelines, integrating data preprocessing and model fitting into a unified workflow. This approach minimized the risk of data leakage and ensured full reproducibility of the analytical process. Model training was carried out exclusively on the training subset, and performance was subsequently assessed using the independent test data.

### 2.8. Performance Metrics

Model performance was evaluated solely on the independent test set to ensure an unbiased assessment of predictive ability. Discriminative power was quantified using the area under the receiver operating characteristic curve (AUC-ROC) for binary classification, and the macro-averaged One-vs-Rest AUC for potential multiclass scenarios. To provide a comprehensive overview of model behavior, additional performance metrics were computed, including accuracy, precision, recall (sensitivity), and F1-score. These complementary indicators allowed for a balanced interpretation of predictive performance across classes.

For each algorithm, individual ROC curves were generated and exported in high-resolution format (600 dpi). A summary figure comparing all ROC curves was also created to visually contrast model performance. When a classifier produced a decision function rather than direct probabilities, a sigmoid transformation was applied to enable ROC curve computation and visualization. This adjustment improved interpretability through probability scaling without affecting the underlying AUC values.

### 2.9. Model Explainability

Model interpretability was assessed using SHapley Additive exPlanations (SHAP) to estimate the contribution of each predictor to the model’s output. For tree-based algorithms, including Random Forest, Gradient Boosting, and Decision Tree models, the TreeExplainer method was employed. In the case of linear models such as Logistic Regression and linear SVMs, the LinearExplainer with interventional perturbation was applied. For nonlinear algorithms lacking native SHAP integration—namely MLP, RBF SVM, kNN, and Naïve Bayes—the KernelExplainer was implemented using a background sample of 100 randomly selected training observations and 200 test instances for visualization purposes.

The interpretability outputs included a series of global and local explanation plots: bar charts of mean absolute SHAP values, beeswarm plots, and dependence plots for the three most influential predictors. Additionally, individual waterfall plots were generated to illustrate subject-specific feature contributions. All figures were exported at 600 dpi resolution. SHAP values were expressed in log-odds, where negative values indicated a reduced probability of survival—reflecting an increased predicted risk of in-hospital mortality, given that the positive class corresponded to survival.

### 2.10. SHAP-Based Risk Equation

For the best-performing model according to AUC, an interpretable risk equation was derived using SHAP values. The model prediction for each individual can be expressed as:

$$logit\left(p\right)=\beta_{0}+\sum_{i}\phi_{i},\;p=\frac{1}{\left(1+\exp\left(-logit\left(p\right)\right)\right)},\;RR=\frac{p}{p^{0}}$$

where β_0_ represents the SHAP base value, corresponding to the expected model output (in log-odds) across the entire dataset, and ϕ_i_ denotes the individual contribution of predictor *i* to the predicted outcome. The baseline probability can be obtained as p_0_ = σ(β_0_), where σ is the logistic (sigmoid) function.

This formulation allows the decomposition of each patient’s predicted risk into additive contributions from all predictors, providing an individualized and transparent estimation of mortality risk directly interpretable in clinical terms.

### 2.11. Handling of Class Imbalance

Given the relatively low prevalence of in-hospital deaths, class imbalance was managed by setting the parameter class_weight = “balanced” in all algorithms that supported this option. This adjustment automatically reweighted the contribution of each class in inverse proportion to its frequency, reducing bias toward the majority (survival) class. In addition, a stratified train–test split was used to maintain the original outcome distribution across both subsets.

To preserve the empirical characteristics and natural class proportions of the dataset, no oversampling or synthetic data generation techniques were applied in the main analysis. This decision ensured that model training reflected the real-world clinical context represented in the national hospitalization data.

### 2.12. Sensitivity Analyses

Several pre-specified sensitivity analyses were performed to evaluate the robustness and clinical interpretability of the developed models. First, the orientation of the positive class was reversed—defining death instead of survival as the reference outcome—to verify the consistency of SHAP values and predicted probabilities. Second, the stability of categorical encoding was examined by consolidating low-frequency categories within the transferring service variable to reduce sparsity effects. Third, the reliability of complex algorithms was tested by varying key hyperparameters, including the regularization parameter C in the Support Vector Classifier and the number of estimators in the Random Forest model.

In addition, several methodological extensions were identified as recommended for future work. These include probability calibration using Platt or isotonic scaling, combined with calibration plots and Brier scores; estimation of 95% confidence intervals for the AUC using bootstrap or DeLong methods; and external validation—temporal or geographic—to assess model generalizability. Decision-curve analysis was also proposed to evaluate clinical usefulness, along with fairness auditing across sex, age, and ethnicity to ensure equitable model performance.

### 2.13. Statistical Considerations

The unit of analysis was the hospitalization episode, with each record corresponding to a distinct patient admission. Records were excluded only when essential variables required for cohort definition or outcome ascertainment, such as age, sex, or discharge status, were missing; missing values in predictor variables were handled using median imputation for continuous variables and mode imputation for categorical variables. Model outputs were primarily descriptive and comparative, and no correction for multiplicity was applied, as all algorithms evaluated the same predictive construct under equivalent analytical conditions. Hospital-level effects were not explicitly modeled, because the analytical focus was placed on patient-level risk prediction and on maximizing generalizability across the national public hospital network. To ensure full reproducibility, the random seed parameter (random_state = 42) was fixed across all stages of the workflow, including data partitioning, model training, and evaluation. Confidence intervals for the AUC were not computed in the main analysis to optimize computational efficiency; however, these can be readily obtained through stratified bootstrap resampling, which yields nonparametric 95% confidence intervals for model discrimination.

### 2.14. Software and Reproducibility

All analyses were conducted in Python 3.12.2 using the Jupyter Notebook environment (version 7.1.0). The computational workflow was built with widely adopted libraries, including pandas for data manipulation, scikit-learn for machine learning pipelines, matplotlib for visualization, SHAP for interpretability, and python-docx for automated report generation.

The analytical script was designed to automatically log diagnostic information—such as delimiter and encoding detection or column mapping—while executing all preprocessing and model training steps. It also computed the full set of performance metrics and exported publication-ready outputs, including metric tables (CSV), high-resolution ROC curves and SHAP visualizations (600 dpi), as well as a structured summary report in DOCX format.

To further strengthen reproducibility, the workflow can be containerized (for instance, using Docker 27) and accompanied by the public release of both the source code repository and a synthetic dataset. The latter should retain the statistical structure of the original data while safeguarding patient privacy.

## 3. Results

The study included 40,253 hospitalization episodes of older adults with hip fracture. The mean age of the cohort was 81.9 years (SD 9.1), and women accounted for 76.8% of the sample. Regarding disease burden and treatment, the mean number of comorbidities was 6.6 (SD 4.2), with the majority of patients presenting three or more comorbid conditions. Most patients underwent surgical treatment during hospitalization (88.0%), while 12.0% were managed non-operatively. The mean length of hospital stay was 12.7 days (SD 13.9).

The Gradient Boosting model demonstrated the best overall performance, achieving an AUC-ROC of 0.885, an F1-score of 0.791, precision of 0.738, and recall of 0.849, indicating a strong balance between sensitivity and specificity (Table 1).

The MLP neural network and linear approaches—Logistic Regression and linear SVM—reached comparable AUC values (approximately 0.88). However, their calibration between precision and recall was less optimal, which may limit their interpretability in clinical settings. In contrast, algorithms such as Random Forest and k-Nearest Neighbors achieved very high overall accuracies (≥0.96) but markedly low F1-scores (≤0.12), suggesting a strong bias toward the majority (survival) class and poor discrimination of deaths. The Naïve Bayes classifier yielded the highest recall (0.989) but an AUC of only 0.499, indicating near-random predictive performance.

**Figure 1:**
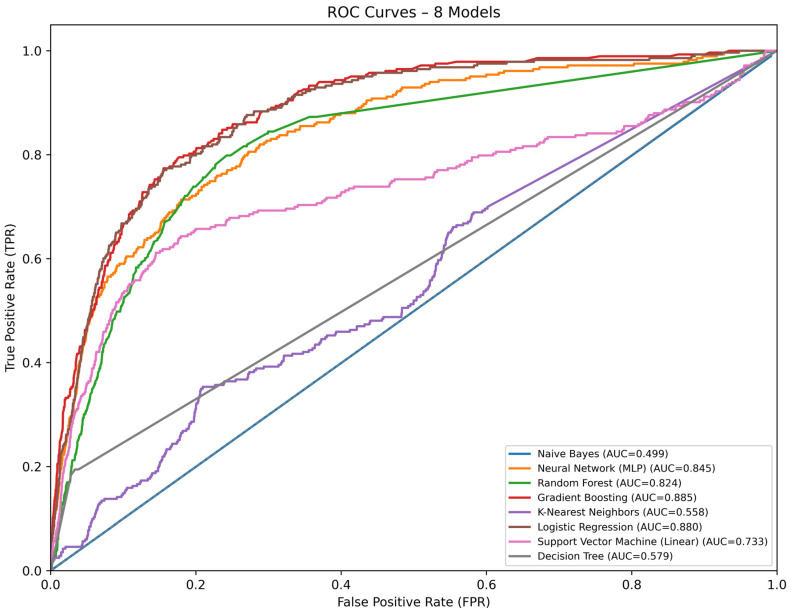
Receiver operating characteristic (ROC) curves for the eight supervised machine learning models. Interpretability analysis was performed using SHapley Additive exPlanations (SHAP) on the Gradient Boosting model. The global importance ranking (Figure 2) revealed that comorbidities had the largest contribution to model output (|SHAP| ≈ 0.52), followed by surgical treatment (|SHAP| ≈ 0.47) and age (|SHAP| ≈ 0.27). Process-related variables such as first hospital service (≈0.08) and length of stay (≈0.07) showed smaller but informative effects, while demographic factors (e.g., sex and ethnicity) had minimal impact (<0.02).

Taken together, the Gradient Boosting algorithm provided the most favorable compromise between discriminative capacity and clinical relevance. Consequently, it was selected for interpretability assessment and individualized mortality risk analysis (Figure 1).

**Figure 3:**
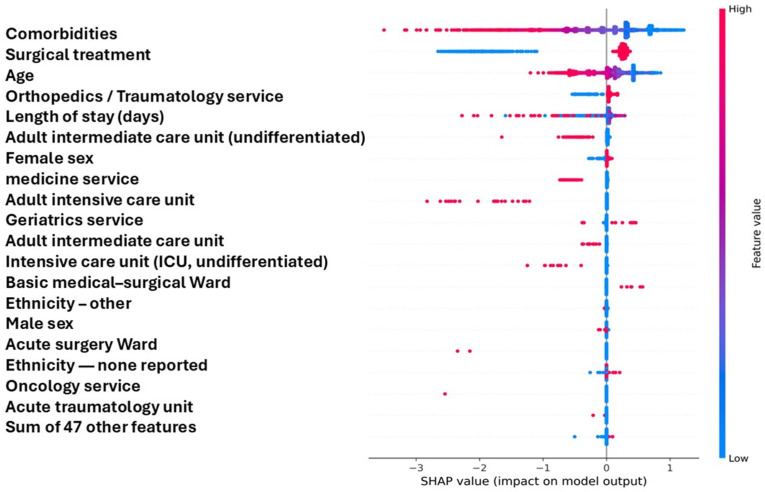
SHAP summary plot showing the impact of each predictor on model output for in-hospital mortality after hip fracture.

The beeswarm plot (Figure 3) illustrated that higher comorbidity counts consistently shifted predictions toward lower survival probability (negative SHAP values), whereas surgical treatment was associated with positive SHAP contributions, indicating a protective effect. Age and prolonged hospitalization also produced predominantly negative shifts, aligning with poorer outcomes. Categories corresponding to intensive or high-complexity care (e.g., ICU or internal medicine) showed small but directionally coherent negative effects.

Dependence plots confirmed these trends:

- Comorbidities showed a monotonic negative gradient, with risk increasing sharply beyond three comorbidities.
- Surgery displayed two distinct clusters consistent with a binary variable, with surgery exerting a stable protective effect across age groups.
- Age demonstrated a nonlinear negative relationship with predicted survival, with steeper declines in older patients.

Collectively, these results indicate that the model’s predictions are driven primarily by disease burden, surgical status, and age—factors that are both clinically plausible and interpretable within the inpatient context.

**Figure 4:**
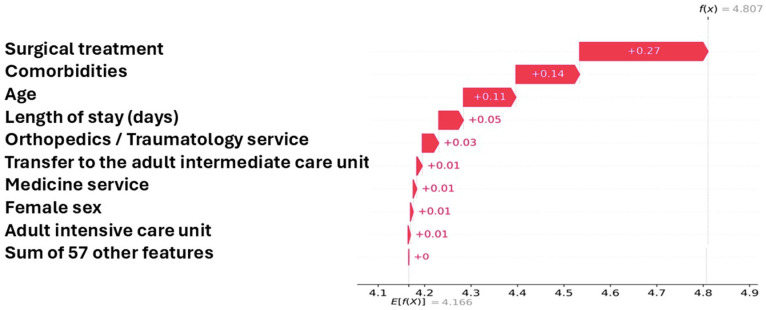
SHAP dependence plot showing the directional impact of top predictors on in-hospital mortality after hip fracture.

Individual SHAP waterfall plots (Figure 4) illustrated how the contribution of each predictor combined to shape the model’s prediction for individual patients. In a representative case with a high predicted probability of survival, the largest positive contributions were associated with surgical treatment (+0.27), a low number of comorbidities (+0.14), and younger age (+0.11), while a minor negative influence was attributed to a longer hospital stay. This additive breakdown shows how SHAP values can be interpreted as shifts in log-odds, providing transparent insight into the model’s reasoning at the individual patient level.

**Figure 5:**
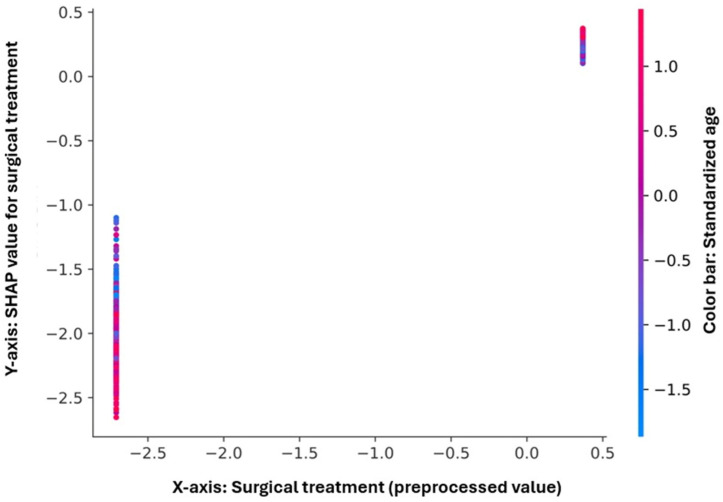
SHAP dependence plot for surgical treatment. The plot depicts the relationship between surgical treatment (*x*-axis, after preprocessing) and its SHAP contribution to the model output (*y*-axis). Two well-defined clusters are observed, corresponding to non-operated and operated patients. Negative SHAP values (left cluster) indicate increased predicted mortality, whereas positive SHAP values (right cluster) correspond to lower mortality risk (protective effect). The color gradient represents standardized age, showing minimal interaction with surgical status. The low within-cluster dispersion suggests a stable and robust influence of surgical treatment on model predictions.

**Figure 6:**
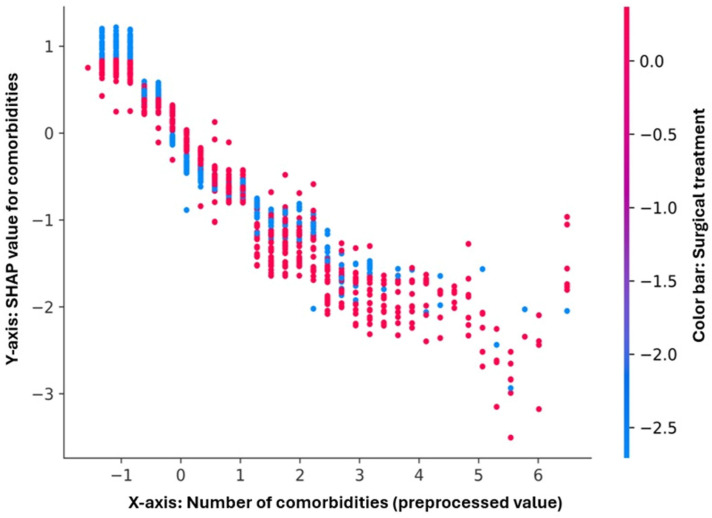
SHAP dependence plot for comorbidities. This plot shows the relationship between the number of comorbidities (*x*-axis, after preprocessing) and their SHAP contribution to the model output (*y*-axis). A clear monotonic downward gradient is observed: higher comorbidity counts are associated with more negative SHAP values, indicating increased predicted mortality risk. The color gradient represents surgical treatment, showing that both operated and non-operated patients follow a similar negative pattern, confirming that comorbidity burden exerts a dominant and consistent adverse effect on model predictions.

**Figure 7:**
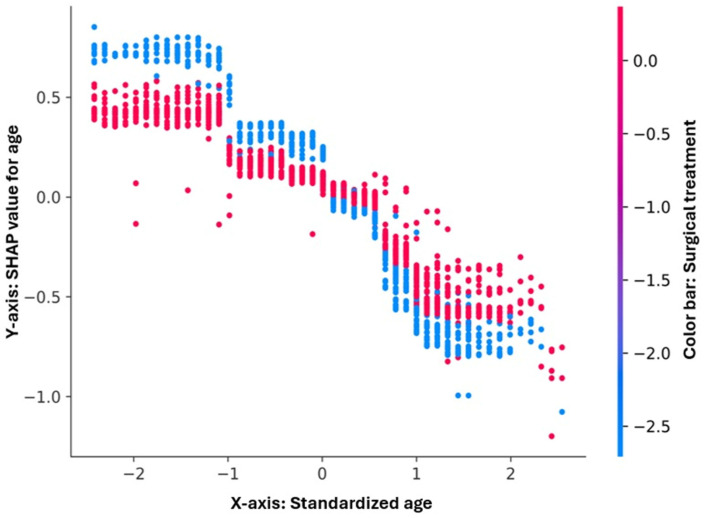
SHAP dependence plot for age. The plot illustrates the relationship between standardized age (*x*-axis) and its SHAP contribution to the model output (*y*-axis). A clear negative gradient is observed, indicating that older patients contribute negatively to the log-odds of survival (higher predicted mortality risk). Younger patients show positive SHAP values, suggesting a protective effect. The color gradient represents surgical treatment, revealing a mild interaction: at similar age levels, operated patients tend to cluster slightly higher on the SHAP scale, consistent with a modest protective influence of surgery. Overall, age emerges as a strong and nonlinear determinant of individual mortality risk predictions.

Dependence plots were created for the three most influential predictors—surgical treatment, number of comorbidities, and age—to illustrate their directional influence on the model’s log-odds predictions (Figure 5, Figure 6 and Figure 7). In the case of surgical treatment (Figure 5), two distinct clusters were observed: non-operated patients exhibited strongly negative SHAP values, reflecting a higher predicted risk of mortality, while operated patients showed positive SHAP contributions, consistent with a protective effect. The color gradient by age revealed only a minor interaction, suggesting that the survival benefit associated with surgery remained stable across different age groups.

The comorbidity burden exhibited a monotonic downward trend in SHAP values (Figure 6). As the number of comorbidities increased, the predicted log-odds of survival decreased, particularly beyond three or four comorbidities, suggesting a threshold effect. This variable showed the strongest and most consistent negative influence, independent of surgical status.

The age variable exhibited a clear nonlinear relationship with the model’s output (Figure 7). Younger patients were associated with positive SHAP values, reflecting a lower predicted risk of in-hospital mortality, whereas advancing age corresponded to progressively negative SHAP contributions. The interaction coloring by surgical treatment showed that the protective effect of surgery persisted across all age groups, although it tended to diminish slightly among the oldest patients.

Taken together, these dependence plots reinforce the clinical plausibility of the model’s predictions, underscoring age, comorbidities, and surgical intervention as the key determinants of in-hospital mortality risk.

Finally, to provide an interpretable quantitative expression of predicted risk, a SHAP-based risk equation was derived from the Gradient Boosting model—the best-performing algorithm according to AUC. The model output can be expressed as a sum of additive feature contributions (ϕ_i_) on the log-odds scale:

$$logit\left(p\right)=\;\beta_{0}+\;\sum_{i}\phi_{i}$$

where β_0_ represents the model baseline (expected log-odds), and ϕ_i_ denotes the SHAP value for predictor *i*. The probability of in-hospital survival or mortality is then obtained as:

$$p\;=\;\frac{1\;}{1\;+\exp\left(-logit\left(p\right)\right)}$$

The relative risk (RR) for an individual patient can be expressed as:

$$RR\;=\;\frac{p}{p^{0}},\;p^{0}=\;\sigma \left(\beta^{0}\right)$$

For the Gradient Boosting model, the estimated baseline value was β_0_ ≈ 4.1660, corresponding to a baseline relative risk RR_0_ ≈ 1.0073. This representation enables the direct computation of individualized mortality risk from the SHAP decomposition, thereby combining interpretability with quantitative precision.

## 4. Discussion

The main finding of this nationwide study is that a Gradient Boosting machine learning model achieved excellent discrimination for predicting in-hospital mortality among older adults hospitalized with hip fracture (AUC-ROC = 0.885), outperforming the other evaluated algorithms. In addition to its predictive performance, the model provided clinically meaningful interpretability through SHAP analysis, identifying comorbidity burden, surgical treatment, and age as the most influential determinants of mortality risk. These results demonstrate that interpretable, data-driven models based on routinely collected hospital data can effectively support early risk stratification in real-world inpatient settings.

Our findings are consistent with previous research that has applied machine learning techniques to predict clinical outcomes in patients with hip fractures. For example, a recent 2024 study from China reported that an Extreme Gradient Boosting (XGBoost) model achieved an AUC of approximately 0.91 for predicting in-hospital mortality after hip surgery, identifying age, comorbidities, and surgical intervention as the most influential variables. These results closely mirror the patterns observed in our study, reinforcing the robustness and generalizability of tree-based ensemble methods for mortality prediction in this population [20]. Similarly, a Thai study using multiple models reported AUCs ranging from 0.81 to 0.99 for one-year mortality prediction, also highlighting the utility of Random Forest and Gradient Boosting techniques [21]. In contrast, a retrospective analysis in Spain with a smaller sample (~500 patients) failed to surpass an AUC of 0.65, underscoring the need for large, well-curated datasets to build robust models [22]. Additionally, a Swedish registry study involving over 124,000 cases found that regularized logistic regression achieved an AUC of ~0.74, showing that in some contexts, traditional statistical models remain competitive, though with more limited interpretability at the individual level [19].

The SHAP analysis provides important interpretative insights into how established clinical predictors contribute to mortality risk after hip fracture. Although age, comorbidity burden, and surgical treatment are well-known determinants of prognosis, SHAP analysis adds value by quantifying their relative and nonlinear contributions at the individual patient level. In particular, nonlinear age effects, threshold patterns in comorbidity burden, and the consistently protective contribution of surgical treatment across age strata were observed. This patient-level risk decomposition extends beyond traditional summary statistics or conventional mortality scores and supports transparent, individualized risk stratification in real-world inpatient settings. Although SHAP values were used to identify the most influential predictors, the models were not refitted using only SHAP-ranked features, as SHAP was applied for interpretability rather than post hoc feature selection. Restricting the feature set based solely on importance rankings may lead to information leakage or overly optimistic performance estimates and may overlook complex interactions captured by the full set of clinically plausible predictors.

Several mortality risk scores have been developed to support prognostic assessment after hip fracture, including the Nottingham Hip Fracture Score and indices based on comorbidity burden such as the Charlson Comorbidity Index. These tools have demonstrated clinical utility and remain widely used in routine practice. Rather than replacing established mortality scores, the present machine learning approach should be viewed as complementary. By integrating routinely collected hospital variables and allowing nonlinear interactions, the Gradient Boosting model achieved higher discriminative performance while providing individualized risk decomposition through SHAP-based explanations. This feature enables transparent interpretation of how traditional risk factors—such as age, comorbidities, and surgical treatment—contribute to predicted mortality risk at the patient level, thereby extending the interpretability of conventional scoring systems.

From a clinical perspective, one of the most relevant findings is the negative impact of the number of comorbidities on survival after hip fracture. This is consistent with the geriatric literature, which has consistently shown that a higher burden of chronic diseases worsens prognosis. For example, each one-point increase in the age-adjusted Charlson Comorbidity Index is associated with approximately a 31% increase in mortality risk among hip fracture patients [23]. Likewise, patients with more than three comorbidities experience substantially higher rates of infectious complications and death within the first year after the fracture compared to those with fewer comorbidities [24,25]. Our results confirm the number of comorbidities as the most important predictor, reflecting how preexisting conditions such as heart disease, renal failure, neurological disorders (e.g., dementia), or cancer reduce the patient’s physiological reserve and hinder recovery after trauma. In fact, in a study focused on octogenarian patients with hip fracture, the Charlson Index was found to be a better predictor of in-hospital mortality than the ASA scale (BMC Surg), underscoring the importance of quantifying multimorbidity [26]. Our findings reaffirm that high comorbidity translates into greater vulnerability and a higher risk of fatal outcomes; therefore, risk stratification should carefully consider this factor and encourage perioperative interventions aimed at optimizing preexisting medical conditions.

Another notable finding is the protective effect of surgical treatment on in-hospital mortality. In our model, patients who underwent surgery had a lower predicted probability of death during hospitalization, a result that aligns with well-established clinical evidence. Most hip fractures in older adults require surgical repair, and numerous studies have shown that early surgical intervention significantly improves survival outcomes. International guidelines, such as those from the American Academy of Orthopaedic Surgeons (AAOS), recommend performing surgery within 48 h of the fracture, as timely intervention reduces both mortality and postoperative complications [27,28]. Conversely, the non-surgical option is typically reserved for extremely frail patients or those with terminal comorbidities for whom surgical risk is prohibitive. Previous studies have shown that non-surgically managed patients have markedly higher mortality rates than those who undergo surgery, although this difference is influenced by the poorer baseline condition of the non-operated group [29]. In our cohort, the higher mortality observed among patients who did not undergo surgery is likely attributable to their older age, a greater proportion of very elderly men, and a higher comorbidity burden compared with those who were operated on, factors that are well recognized to increase mortality risk following hip fracture. Consequently, the apparent protective effect of surgery reflects not only the intrinsic benefits of fracture repair—such as enabling early mobilization, effective pain control, and prevention of complications like thrombosis or pressure ulcers—but also a degree of selection bias, since only patients deemed clinically stable are typically eligible for surgery. Nevertheless, our findings reinforce current clinical recommendations to provide surgical treatment to all appropriate candidates, accompanied by adequate preoperative optimization. Timely surgery remains a modifiable determinant of survival and should be prioritized as a cornerstone of hip fracture management in older adults.

Advanced age also emerged as an important prognostic factor, in line with clinical experience and epidemiological studies. As patient age increases, so does the risk of mortality after a hip fracture; it has been estimated that mortality rates may rise by up to 30% in the oldest groups compared with younger adults [30]. This effect is attributed to the biological frailty associated with aging: very old adults often present sarcopenia, reduced cardiac and respiratory reserve, immune alterations, and higher susceptibility to adverse events such as delirium or infections, all of which contribute to worse outcomes. It is therefore unsurprising that age is included in traditional prognostic scales (such as the Nottingham Hip Fracture Score) and in indices like Charlson, and that it consistently appears among mortality predictors identified in modern analyses [19,31]. In the study by Forssten et al., for instance, advanced age was one of the main factors associated with increased one-year mortality [19]. In our cohort, we observed the same trend: older patients had lower in-hospital survival rates. This highlights the need for comprehensive geriatric approaches for very old hip fracture patients, including full geriatric assessment, nutritional optimization, delirium prevention, and aggressive management of acute comorbidities, with the goal of mitigating the negative impact of age on prognosis.

Finally, other findings of our study deserve mention for their clinical relevance, even if they carried a smaller weight in the predictive model. We observed that prolonged hospitalization tends to be associated with worse outcomes. This is consistent with previous reports linking longer hospital stays to higher short-term post-discharge mortality [32]. A plausible explanation is that prolonged hospital stays often arise from medical complications occurring during hospitalization—such as nosocomial infections, pneumonia, thromboembolism, or delirium—or from delayed functional recovery. These complications, in turn, substantially increase the risk of death. Consequently, reducing the length of stay through early rehabilitation, infection prevention, and proactive management of comorbid conditions could have a meaningful impact on survival outcomes. Another relevant aspect is the type of hospital service and care model. The literature has shown that implementing shared-care models between orthopedic surgeons and geriatricians (orthogeriatric units) is associated with lower in-hospital mortality among hip fracture patients [29]. Orthogeriatric collaboration ensures more holistic care: it optimizes comorbidity management upon admission, tailors surgical indication and timing to patient conditions, and promotes preventive interventions (early mobilization, nutritional support, avoidance of inappropriate polypharmacy) that impact recovery. In our setting, adopting a similar multidisciplinary approach could partly explain the favorable outcomes observed and represents an opportunity for improvement where such models are not yet implemented. In summary, these findings reaffirm that mortality following hip fracture in older adults is a multifactorial phenomenon determined by the interaction between patient baseline characteristics (age, comorbidities, functional status), treatment factors (performance and timing of surgery), and the quality of hospital care (prevention of complications, interdisciplinary management). Addressing each of these components comprehensively is essential to reduce mortality and improve prognosis in this vulnerable population [29,32].

From a clinical perspective, the proposed Gradient Boosting model may have several practical applications in the inpatient management of older adults with hip fracture. By leveraging routinely available hospital data, the model could support early risk stratification at admission, helping clinicians identify patients at higher risk of in-hospital mortality who may benefit from closer monitoring, multidisciplinary care, or early optimization of comorbid conditions. In particular, the strong contribution of comorbidity burden, surgical treatment, and age aligns with current orthogeriatric principles and reinforces the importance of timely surgical decision-making and comprehensive medical management. Moreover, the use of SHAP-based explanations enables transparent, patient-level interpretation of risk, facilitating clinical communication and supporting shared decision-making without replacing established clinical judgment or existing mortality risk scores.

This study has several limitations that warrant consideration. First, it relied on a retrospective cohort derived from a single national health system (FONASA), which may limit the generalizability of the findings to other healthcare settings. Although the dataset encompasses a large proportion of the Chilean population, external validation—either temporal or geographic—was not performed in the present analysis due to reliance on a single nationwide administrative database. Second, the set of predictors was restricted to administrative and care-related variables routinely available in hospital discharge records. The absence of physiological measures (e.g., vital signs), laboratory results, and detailed perioperative indicators (e.g., ASA score, time to surgery) may have introduced residual confounding and reduced the precision of individual risk estimation. Third, the outcome definition—in-hospital death at discharge—does not account for deaths occurring shortly after discharge. As a result, time-to-event or competing-risk models could not be applied, potentially leading to an underestimation of early post-discharge mortality. Finally, it is important to note that SHAP-based explanations describe model-derived associations, not causal relationships. Although SHAP enhances model transparency and interpretability, its results should be understood within the framework of predictive inference rather than causal explanation.

## 5. Conclusions

In this nationwide cohort of older adults hospitalized with hip fracture, a Gradient Boosting machine learning model demonstrated excellent discrimination for predicting in-hospital mortality using routinely collected hospital data. Comorbidity burden, surgical treatment, and age emerged as the most influential predictors of mortality risk. The integration of SHAP analysis enhanced model transparency by providing clinically interpretable, patient-level explanations. These findings support the potential role of interpretable machine learning models as complementary tools for early risk stratification in real-world inpatient settings. Future studies should focus on external validation and evaluation of clinical impact.

## Figures and Tables

**Figure 2:**
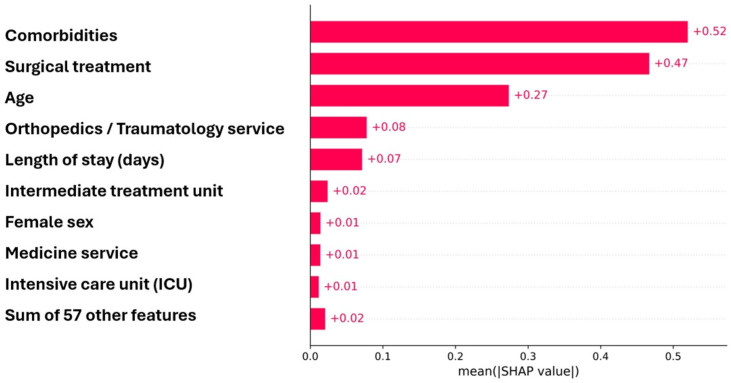
Mean absolute SHAP value (|SHAP|) contribution of predictors for in-hospital mortality after hip fracture.

**Table 1:** Performance metrics of supervised machine learning models for in-hospital mortality prediction.

Model	Accuracy	Precision	Recall (Sensitivity)	F1-Score	AUC-ROC
Gradient Boosting	0.965	0.738	0.849	0.791	0.885
Multilayer Perceptron (Neural Network)	0.965	0.486	0.060	0.107	0.884
Logistic Regression	0.825	0.141	0.777	0.238	0.880
Linear Support Vector Machine	0.832	0.145	0.770	0.244	0.879
Random Forest	0.957	0.218	0.085	0.122	0.824
k-Nearest Neighbors	0.960	0.253	0.078	0.119	0.697
Decision Tree	0.935	0.156	0.194	0.173	0.579
Gaussian Naïve Bayes	0.043	0.035	0.989	0.068	0.499

## Data Availability

The raw data supporting the conclusions of this article will be made available by the authors on request.

## Abbreviations

The following abbreviations are used in this manuscript:

AAOS	American Academy of Orthopaedic Surgeons
AI	Artificial intelligence
ASA	American Society of Anesthesiologists physical status classification
AUC-ROC	Area under the receiver operating characteristic curve
FONASA	Fondo Nacional de Salud (National Health Fund, Chile)
ICD-10	International Classification of Diseases, Tenth Revision
ICU	Intensive care unit
kNN	k-Nearest Neighbors
MLP	Multilayer Perceptron
RBF	Radial basis function
ROC	Receiver operating characteristic
RR	Relative risk
SHAP	Shapley Additive Explanations
SVM	Support Vector Machine
YLDs	Years lived with disability
